# Transmitter inputs to different motoneuron subgroups in the oculomotor and trochlear nucleus in monkey

**DOI:** 10.3389/fnana.2015.00095

**Published:** 2015-07-24

**Authors:** Christina Zeeh, Michael J. Mustari, Bernhard J. M. Hess, Anja K. E. Horn

**Affiliations:** ^1^Institute of Anatomy and Cell Biology, Department I, Ludwig-Maximilians UniversityMunich, Germany; ^2^Washington National Primate Research Center and Department of Ophthalmology, University of WashingtonSeattle, WA, USA; ^3^Vestibulo-Oculomotor Laboratory Zürich, Department of Neurology, University HospitalZürich, Switzerland

**Keywords:** Glycine, GABA, vGlut, C-group, extraocular muscles

## Abstract

In all vertebrates the eyes are moved by six pairs of extraocular muscles enabling horizontal, vertical and rotatory movements. Recent work showed that each extraocular muscle is controlled by two motoneuronal groups: (1) Motoneurons of singly-innervated muscle fibers (SIF) that lie within the boundaries of motonuclei mediating a fast muscle contraction; and (2) motoneurons of multiply-innervated muscle fibers (MIF) in the periphery of motonuclei mediating a tonic muscle contraction. Currently only limited data about the transmitter inputs to the SIF and MIF motoneurons are available. Here we performed a quantitative study on the transmitter inputs to SIF and MIF motoneurons of individual muscles in the oculomotor and trochlear nucleus in monkey. Pre-labeled motoneurons were immunostained for GABA, glutamate decarboxylase, GABA-A receptor, glycine transporter 2, glycine receptor 1, and vesicular glutamate transporters 1 and 2. The main findings were: (1) the inhibitory control of SIF motoneurons for horizontal and vertical eye movements differs. Unlike in previous primate studies a considerable GABAergic input was found to all SIF motoneuronal groups, whereas a glycinergic input was confined to motoneurons of the medial rectus (MR) muscle mediating horizontal eye movements and to those of the levator palpebrae (LP) muscle elevating the upper eyelid. Whereas SIF and MIF motoneurons of individual eye muscles do not differ numerically in their GABAergic, glycinergic and vGlut2 input, vGlut1 containing terminals densely covered the supraoculomotor area (SOA) targeting MR MIF motoneurons. It is reasonable to assume that the vGlut1 input affects the near response system in the SOA, which houses the preganglionic neurons mediating pupillary constriction and accommodation and the MR MIF motoneurones involved in vergence.

## Introduction

The vertebrate eye is rotated by six extraocular muscles: four recti (superior, inferior, medial and lateral recti muscles) and two oblique muscles (superior and inferior oblique). The muscles are innervated by motoneurons lying in the tegmentum of the brainstem. Motoneurons of the oculomotor nucleus (nIII) innervate the ipsilateral medial rectus (MR), inferior rectus (IR), inferior oblique (IO) and contralateral superior rectus (SR) muscles. Motoneurons of the trochlear nucleus (nIV) control the contralateral superior oblique muscle (SO), and motoneurons of the abducens nucleus (nVI) activate the ipsilateral lateral rectus (LR) muscle (Büttner-Ennever, 2006). The levator palpebrae (LP) motoneurons lie in a separate cluster at the midline in caudal nIII termed the central caudal nucleus (CCN; Porter et al., 1989).

Each eye muscle has a highly complex morphology and consists of at least six different muscle fiber types, which can be divided into two main categories. Firstly, there are slowly contracting (non-twitch) muscle fibers innervated by multiple “en grappe” endplates that are distributed along the whole muscle fiber (multiply-innervated fibers, MIF). Secondly, there are fast contracting (twitch) muscle fibers innervated by one single “en plaque” ending in the middle third of the muscle fiber (singly-innervated fibers, SIF; Chiarandini and Stefani, 1979; Lynch et al., 1994; for review: Spencer and Porter, 2006). Tract-tracing experiments in monkey and rat revealed that the MIF and SIF motoneurons of all eye muscles form anatomically separated populations. SIF motoneurons lie within the boundaries of the classical motonuclei (nIII, nIV, nVI), whereas the MIF motoneurons appear in subgroups in the periphery of the motonuclei (Büttner-Ennever et al., 2001; Eberhorn et al., 2005). Thereby, in monkey the MIF motoneurons of the MR and IR are situated together in the C-group at the dorsomedial border of nIII. Those of IO and SR are located midline within the S-group sandwiched between the two oculomotor nuclei. The MIF motoneurons of the SO form a dorsal cap of nIV, and those of the LR are arranged as a shell around the medial and ventral aspect of nVI (Büttner-Ennever et al., 2001). Recent studies in monkey revealed that neurons within these peripheral cell groups also give rise to the palisade endings located at the myotendinous junctions of MIFs (Lienbacher et al., 2011; Zimmermann et al., 2011).

Experiments injecting retrograde transsynaptic tracers into monkey eye muscles revealed that SIF and MIF motoneurons receive inputs from different premotor neurons subserving different functions. Whereas SIF motoneurons are targeted by premotor afferents involved in the generation of eye movements, e.g., saccadic burst neurons, secondary vestibulo-ocular neurons, the peripheral MIF motoneurons are targeted mainly by afferents from premotor sources involved in gaze holding (Wasicky et al., 2004; Ugolini et al., 2006).

Significant progress has been made in the histochemical characterization of premotor inputs to motoneurons of individual extraocular eye muscles (for review: McElligott and Spencer, 2000; Horn, 2006; Sekirnjak and du Lac, 2006). These inputs differ in several points, one of them being the selective association of the calcium-binding protein calretinin (CR) with nerve endings targeting motoneurons involved in upgaze (Zeeh et al., 2013). Monkey studies with different methodical approaches suggest that GABA is the major inhibitory neurotransmitter of premotor neurons involved in vertical eye movements, whereas glycine acts as inhibitory transmitter of premotor neurons mediating horizontal eye movements (Spencer et al., 1989, 1992; Spencer and Baker, 1992). So far, few attempts have been made to study differing transmitter-related inputs to MIF vs. SIF motoneurons (Ying et al., 2008). In the present study we investigated the presence of glycinergic, GABAergic and glutamatergic inputs to SIF and MIF motoneurons of nIII and nIV in monkey. Preliminary results have been reported in abstract form (Schulze et al., 2009).

## Materials and Methods

The tracer injections were undertaken either at the Department of Neurology at the University Hospital in Zürich (case 2) or at the National Primate Research Center at the University of Washington in Seattle (case 1). All experimental procedures conformed to the state and university regulations for laboratory animal care, including the Guide Principles of Laboratory Animal Care (NIH 8^th^ edition, revised 2011) and they were approved by animal care officers and the institutional Animal Care and Use Committees. The surgical procedures for tracer-injections into the extraocular muscle were described in detail in a previous report (Büttner-Ennever et al., 2001). All experimental cases are listed in Table 1.

**Table 1:** **An overview of injection, fixation and immunohistochemistry details for each case**.

Case	Injection	Fixation	Sections	Immunhistochemistry
1	3 μl CTB, MR	4% paraformaldehyde	Frozen	CTB, CTB + GAD, CTB + vGlut1
2	5 μl CTB, MR	4% paraformaldehyde	Frozen	CTB, CTB + GAD
3		4% paraformaldehyde	Paraffin	GAD, vGlut1 or vGlut2 + ChAT
4		4% paraformaldehyde	Frozen	GABA-A +ChAT, GlyT2
5		4% paraformaldehyde	Frozen	GlyR
6		4% paraformaldehyde	Frozen	GlyT2
7		1% paraformaldehyde 2, 5% glutaraldehyde	Frozen	GABA
8		4% paraformaldehyde 0, 3% glutaraldehyde	Frozen	GABA
9		4% paraformaldehyde	Frozen	vGlut2
10	WGA-HRP	4% paraformaldehyde	Frozen	

*CTB, cholera toxin subunit B; GAD, glutamate decarboxylase; vGlut, vesicular glutamate transporter; ChAT, choline acetyltransferase; GABA, gamma-aminobutyric acid, GABA-A, GABA receptor; GlyT, glycine transporter; GlyR, glycine receptor; MR, medial rectus muscle; WGA-HRP, wheat germ agglutinin conjugated to horseradish peroxidase*.

To identify the MR MIF motoneurons prior to glutamate decarboxylase (GAD) or vesicular glutamate transporters (vGlut) immunostaining, two macaque monkeys (case 1, case 2) received a tracer injection of cholera toxin subunit B (CTB) into the MR of the left eye. Each monkey was therefore sedated with Ketamine (Ketalar 1–2 mg/kg) and kept in a surgical plane of anesthesia using Isoflurane inhalation. Under sterile conditions, the MR of the left eye was exposed by retracting the eye lid and by making a conjunctival incision. Volumes of 5 μl (case 1) and 3 μl (case 2) of CTB (1% in aqua bidest) were injected into the myotendinous junctions of the left MR. For post-operative treatment the monkeys received antibiotics and analgesics.

After a survival time of 4 days, the monkeys were euthanized with an overdose of sodium-pentobarbital (80 mg/kg body weight, Merial, Halbergmoos, Germany). Then, the animals were transcardially perfused with 0.9% saline followed by either 4% paraformaldehyde in 0.1 M phosphate buffer or a mixture of 1% paraformaldehyde and 2.5% glutaraldehyde (for GABA staining) in 0.1 M phosphate buffer. Paraformaldehyde fixed brain tissue of five additional monkeys (case 3, case 4, case 5, case 6, case 9) and glutaraldehyde fixed brain tissue of two monkeys (case 7, case 8), all from other projects without eye muscle injections, were used for immunohistochemical staining of transmitter-related proteins only. The brains were removed from the skull and immersed in 10% sucrose in 0.1 M phosphate buffer and transferred to 30% sucrose for frozen sectioning. Alternatively, one 4% paraformaldehyde-fixed brain was embedded in paraffin.

**Figure 1:**
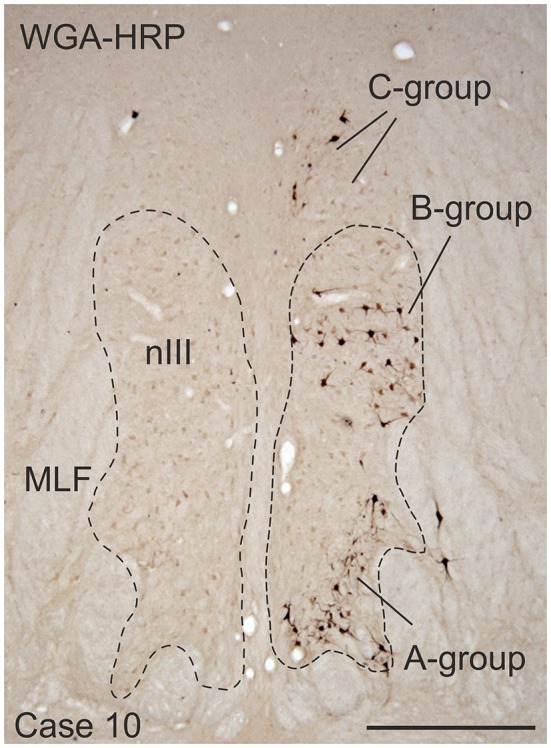
**Coronal section through the oculomotor nucleus (nIII) of a monkey, who had received an injection with wheat germ agglutinin-horseradish peroxidase (WGA-HRP) into the medial rectus (MR) muscle of the right eye**. Retrogradely labeled neurons are found at three locations: ventral in the A-group, dorsolateral in the B-group and in the periphery dorsomedial to nIII in the C-group.Within the C-group motoneurons of multiply-innervated muscle fibers (MIF) in the MR are located in the medial part. Scale bar = 500 μm.

Frozen sections of the brainstems were cut at 40 μm in the transverse stereotaxic plane using a cryostat (MICROM HM 560) and collected free-floating in cold 0.1 M phosphate buffer (pH 7.4). The paraffin block was cut at 10 μm using a sliding microtome (Leica, SM 2000 R) and mounted on superfrost slides (Thermo Scientific, Menzel-Gläser Superfrost Plus). A case from a previous study was used to demonstrate the location of MR SIF and MIF motoneurons (case 10) in Figure 1 (Büttner-Ennever et al., 2001).

### Immmunocytochemical Labeling

Immunohistochemistry was performed on cryo-sections (free-floating) or on paraffin-sections (on slide) applying the antibodies directed against the following antigens: GABA (mAB93), glycine transporter 2 (GlyT2), glycine receptor 1 (GlyR1). On selected sections the motoneurons were identified with the cholinergic marker anti-choline acetyltransferase (ChAT) and combined with immunostaining for either: (1) GABA-A receptor (GABA-A); (2) glutamate decarboxylase (GAD); (3) vesicular glutamate transporter 1 (vGlut1); or (4) vesicular glutamate transporter 2 (vGlut2). An overview of all antibodies with dilutions is given in Table 2.

**Table 2:** **An overview of the primary antibodies and dilutions used for immunolabeling**.

Antibody	Host	Antigen	Manufacturer	Cat. No.	Dilution
GABA-A	Mouse	GABA-A receptor, beta-chain	Chemicon, now Millipore, Billerica, USA	MAB341	1:1000
GABA93 MAb	Mouse	GABA-glutaraldehyde-BSA conjugate	Holstein et al. (2004)	Holstein G, Mt. Sinai, Hospital, New York	1:3000
GAD	Mouse	Glutamate decarboxylase	Biotrend, Cologne, Germany	GC3108	1:4000
GAD_65/67_	Rabbit	Glutamate decarboxylase	Millipore; Billerica, USA	AB1511	1:500 (fluorescence)
GlyT2	Sheep	Glycine transporter 2 (neuronal)	Millipore; Billerica, USA	AB1771	1:5000
GlyR	Mouse	Glycine receptor alpha-1-subunit	Synaptic systems, Goettingen, Germany	146 111	1:1000
ChAT	Goat	Choline acetyltransferase human placental enzyme	Millipore, Billerica, USA	AB144P	1:100
CTB	Goat	Choleragenoid	List Biological Laboratories, Campbell, California	703	1:20,000 1:5000 (fluorescence)
vGlut1	Rabbit	Vesicular glutamate transporter 1	Synaptic systems, Goettingen, Germany	135303	1:3000 1:1000 (fluorescence)
vGlut2	Rabbit	Vesicular glutamate transporter 2	Synaptic systems, Goettingen, Germany	135402	1:500

## Antisera

### Cholera Toxin Subunit B (CTB)

The polyclonal goat anti-choleragenoid antibody (703, LOT 10327A4A, List Laboratories Inc., Campbell, CA, USA) was used to detect the tracer CTB (103B, List) provided by the same manufacturer. This tracing and detection method has been successfully applied in numerous previous studies (e.g., Büttner-Ennever et al., 2001).

### GABA93 MAb

A monoclonal antibody against GABA (GABA93 MAb) was used for the detection of GABA. The specificity of GABA 93 MAb has been published previously (Holstein et al., 2004).

### GABA-A Receptor (GABA-A)

For the detection of GABA-A receptors, we used a monoclonal antibody directed against the beta-chain of the GABA-A receptor (MAB341; formerly Roche 1381458, LOT 0612047758, Clone BD17, Chemicon now part of Milipore, Billerica, MA, USA; Bedford et al., 2001). This antibody is purified from GABA benzodiazepine receptor from bovine cortex.

### Glutamate Decarboxylase (GAD)

Alternatively, GABAergic terminals were detected with a mouse monoclonal antibody against the GABA-synthetizing enzyme GAD (GAD_65/67_ GC3108, batch number Z05507, clone 1111, Biotrend, Cologne, Germany) or the rabbit polyclonal antibody against glutamate decarboxylase 65&67 (AB1511, LOT NG17374444, Millipore, Billerica, MA, USA). This antibody is derived from a synthetic peptide from the carboxy-terminus as predicted from the cloned rat GlyT2.

### Glycine Receptor (GlyR)

A mouse monoclonal antibody against the glycine-receptor was used to detect its localization (146 111, clone mAb2b (GlyR2b), Synaptic Systems, Goettingen, Germany). This antibody mAb2b specifically binds to the N-terminus of the alpha-1-subunit of the glycine receptor (Lorenzo et al., 2006).

### Vesicular Glutamate Transporters (vGluts)

Two different types of vGluts were detected in the study: vGlut1 and vGlut2.

For vGlut1 rabbit polyclonal antibodies were used (1350303, Synaptic Systems, Goettingen, Germany) that were generated against fusion proteins containing glutathione-S-transferase and carboxy-terminal and vGlut1 specific peptides (Bellocchio et al., 1998; Takamori et al., 2000). For the immunolabeling of vGlut2, a rabbit polyclonal antibody was used (8135402, Synaptic Systems, Goettingen, Germany). This antibody was developed against fusion proteins containing glutathione-S-transferase and fragments from the carboxy-terminus of rat vGlut2 (Fremeau et al., 2001; Takamori et al., 2001).

### Choline Acetyltransferase (ChAT)

Cholinergic motoneurons were detected with a polyclonal antibody against ChAT raised in goat (AB144P, LOT LV1583390, Millipore, Billerica, MA, USA). The antibody is directed against the whole enzyme isolated from human placenta, which is identical to the brain enzyme (Bruce et al., 1985).

### Controls

Controls for each primary antibody were carried out by the omission of primary antibodies, which in each case led to unstained sections.

### Deparaffination Procedure

Paraffin embedded sections were dewaxed in three changes of xylene for 5, 15 and 30 min, respectively. Sections were rehydrated in decreasing concentration of alcohol and then rinsed in distilled water for 10 min. For antigen demasking the sections were reacted in 0.01M sodium citrate buffer (pH 8.5–9) at +80°C in a waterbath for 15 min. Then, sections in citrate buffer were allowed to cool down to room temperature for 15 min, rinsed shortly in distilled water and transferred to Tris buffered saline (TBS; pH 7.6) for subsequent immunostaining (Jiao et al., 1999).

### Visualization of the Tracer

To localize the tracer, brainstem sections were immunohistochemically stained with a polyclonal goat antibody against CTB (1:20,000; List Biological laboratories, 703) as described previously (Eberhorn et al., 2006). The antigenic sites were visualized with a reaction in 0.025% diaminobenzidine and 0.015% H_2_O_2_ in 0.1 M TBS (pH 7.6) for 10 min.

### Combined Immunoperoxidase Labeling for Tracer and Different Markers

In selected frozen sections combined immunoperoxidase labeling was used to simultaneously detect the tracer CTB and either GAD or vGlut1. All sections were washed in 0.1 M TBS (pH 7.4) and treated with 1% H_2_O_2,_ in 0.1 M TBS for 30 min to suppress endogenous peroxidase activity. The sections were blocked with 5% normal horse serum in 0.1 M TBS, pH 7.4, containing 0.3% Triton X-100 (Sigma, St. Louis, MO, USA) for 1 h, and subsequently processed with either rabbit anti-vGlut1 (1:3000, Synaptic Systems, 135003) or mouse anti-GAD (1:4000, Biotrend GC 3108) in TBS with 5% normal horse serum and 0.3% Triton X-100 for 48 h at room temperature. After several buffer washes in 0.1 M TBS, the sections were incubated in biotinylated horse anti-rabbit (for vGlut1 1:200; Vector laboratories, Burlingame, CA, USA) or biotinylated horse anti-mouse (for GAD 1:200; Vector laboratories, Burlingame, CA, USA) in 0.1 M TBS (pH 7.4) containing 2% bovine serum albumin for 1 h at room temperature. Following three buffer washes, all sections were incubated in ExtrAvidin-peroxidase (avidin conjugated horseradish peroxidase, 1:1000; Sigma, St. Louis, MO, USA) for 1 h at room temperature. After two rinses in 0.1 M TBS, pH 7.4, and one rinse in 0.05 M TBS, pH 7.6, the antigenic sites were visualized by a reaction in 0.025% DAB, 0.2% ammonium nickel sulfate (Riedl-De Haën; Germany) and 0.015% H_2_O_2_ in 0.05 M TBS (pH 7.6) for 10 min, which yielded a black reaction-product. For the detection of CTB the sections were immunocytochemically treated with anti-CTB (1:20,000, List Biological Laboratories, 703) and visualized with a reaction in 0.025% diaminobenzidine and 0.015% H_2_O_2_ in 0.1 M TBS (pH 7.6) for 10 min which yielded a brown-reaction product as described above. After washing, the sections were mounted, air-dried, dehydrated in alcohol and cover-slipped with DPX (Sigma, St. Louis, MO, USA).

### Combined Immunofluorescence Labeling for Tracer and Different Markers

Selected frozen sections were immunostained for the simultaneous detection of CTB and GAD or vGlut1. After a pretreatment with 5% normal donkey serum in 0.3% Triton X-100 (Sigma, St. Louis, MO, USA) in 0.1 M TBS (pH 7.4) at room temperature for 1 h sections were incubated in a cocktail containing goat anti-CTB (1:5000, List Biological Laboratories, 703) and either rabbit anti-GAD_65/67_ (1:500, Millipore, AB1511) or rabbit anti-vGlut1 (1:1000, Synaptic Systems, 135303) in 5% normal donkey serum with 0.3% Triton X-100 in 0.1 M TBS (pH 7.4) at 4°C for 48 h. After three washes in TBS, sections were treated with a cocktail containing Cy^3^-tagged donkey anti-rabbit (1:200, Dianova, Jackson Immuno Research, Baltimore, MA, USA) and Alexa-488 tagged donkey anti-goat (1:200; Molecular Probes, OR, USA) in 0.1 M TBS (pH 7.4) and 2% bovine serum albumin for 2 h at room temperature. After several buffer rinses free-floating frozen sections were mounted on glass slides and dried at room temperature. Sections were cover-slipped with GEL/MOUNT permanent aqueous mounting medium (Biomeda, CA, USA) and stored in the dark at 4°C.

### Single Immunoperoxidase Labeling for Transmitter and Transmitter Related Proteins

Frozen or paraffin sections were immunocytochemically treated with antibodies against one of the following antigens: GABA (93MAb), glycine transporter 2 (GlyT2), glycine receptor (GlyR) or vesicular glutamate transporter 2 (vGlut2). All sections were washed in 0.1 M TBS (pH 7.4) and then pretreated with 1% H_2_O_2_ in 0.1 M TBS for 30 min and thoroughly washed. The sections were then blocked with either 5% normal horse serum (for GABA or GlyR) or 5% normal rabbit serum (for GlyT2) or 5% normal goat serum (for vGlut2) in 0.1 M TBS, pH 7.4 containing 0.3% Triton X-100 (Sigma, St. Louis, MO, USA) for 1 h. This was followed by an incubation in either mouse anti-GABA (1:3000, Holstein), mouse anti-GlyR1 (1:1000, Synaptic Systems 146 111) in TBS with 5% normal horse serum and 0.3% Triton X-100 or sheep anti-GlyT2 (1:5000, Millipore AB1771) in TBS with 5% normal rabbit serum and 0.3% Triton X-100 or rabbit anti-vGlut2 (1:500, Synaptic Systems 135402) in TBS with 5% normal goat serum and 0.3% Triton X-100 at room temperature for 48 h. After several buffer washes in 0.1 M TBS the sections were incubated in either biotinylated horse anti-mouse IgG (1:200; Vector laboratories, Burlingame, CA, USA; for GABA or GlyR) or biotinylated rabbit anti-sheep (1:200; Vector laboratories, Burlingame, CA, USA; for GlyT2) or biotinylated goat anti-rabbit (1:200; Vector laboratories, Burlingame, CA, USA; for vGlut2) in TBS containing 2% bovine serum albumin at room temperature for 1 h. Antigenic sites were detected after incubation in ExtrAvidin-peroxidase (avidin conjugated horseradish peroxidase, 1:1000; Sigma, St. Louis, MO, USA) and subsequent reaction in 0.025% diaminobenzidine and 0.015% H_2_O_2_ in 0.05 M TBS (pH 7.6) for 10 min to yield a brown reaction product (see above). For vGlut2 the antigenic sites were visualized with a reaction in 0.025% diaminobenzidine, 0.2% ammonium nickel sulfate (Riedl-De Haën; Germany) and 0.015% H_2_O_2_ in 0.05 M Tris-buffer (pH 7.6) for 10 minto yield a black reaction-product. After washing, the sections were mounted, air-dried, dehydrated in alcohol and cover-slipped with DPX (Sigma, St. Louis, MO, USA).

### Combined Immunoperoxidase Labeling for ChAT and Different Markers

In selected frozen and paraffin sections, combined immunoperoxidase labeling served to simultaneously detect ChAT and either GABA-A, GAD, vGlut1 or vGlut2.

Therefore the sections were washed in 0.1 M TBS (pH 7.4) and then pretreated with 1% H_2_O_2_ in 0.1 M TBS for 30 min. After washing, the sections were blocked with 5% normal horse serum in 0.1 M TBS, pH 7.4, containing 0.3% Triton X-100 (Sigma, St. Louis, MO, USA) for 1 h and subsequently processed with mouse antibodies against either GABA-A receptor (1:1000, Chemicon, now Millipore, MAB341) or GAD (1:4000, Biotrend, Cologne, Germany GC3108) or with rabbit antibodies against either vGlut1 (1:3000, Synaptic Systems, 135303) or vGlut2 (1:500, Synaptic Systems, 135402) in TBS with 5% normal horse serum and 0.3% Triton X-100 at room temperature for 48 h. After several buffer washes in 0.1 M TBS, the sections were incubated in biotinylated horse anti-mouse IgG (1:200; Vector laboratories, Burlingame, CA, USA; for GABA-A receptor and GAD) or biotinylated horse anti-rabbit IgG (1: 200; Vector laboratories, Burlingame, CA, USA; for vGlut1 and vGlut2) in TBS containing 2% bovine serum albumin for 1 h at room temperature. After several buffer washes and an 1 h incubation in ExtrAvidin-peroxidase (avidin conjugated horseradish peroxidase, 1:1000; Sigma, St. Louis, MO, USA) at room temperature antigenic sites were detected with 0.025% diaminobenzidine, 0.2% ammonium nickel sulfate (Riedl-De Haën; Germany) and 0.015% H_2_O_2_ in 0.05 M TBS (pH 7.6) for 10 min to yield a black reaction-product.

For the subsequent detection of ChAT, sections were thoroughly washed and incubated in 1% H_2_O_2_ in 0.1 M TBS for 30 min to block residual peroxidase activity. Then, the sections were incubated in 5% normal horse serum in 0.1 M TBS, pH 7.4, containing 0.3% Triton X-100 (Sigma, St. Louis, MO, USA) for 1 h, and treated with goat anti-ChAT (1:100, Millipore AB144P) in 0.1 M TBS, pH 7.4, containing 0.3% Triton X-100 for 48 h at room temperature. After washing in 0.1 M TBS, the sections were incubated in biotinylated horse anti-goat IgG (1:200; Vector laboratories, Burlingame, CA, USA) in TBS containing 5% bovine serum albumin for 1 h at room temperature. The antigen binding sides were detected by incubating sections in ExtrAvidin-peroxidase (avidin conjugated horseradish peroxidase, 1:1000; Sigma, St. Louis, MO, USA) and a subsequent reaction with 0.025% diaminobenzidine and 0.015% H_2_O_2_ in 0.05 M TBS (pH 7.6) for 10 min to yield a brown staining. After washing, the sections were mounted, air-dried, dehydrated in alcohol and cover-slipped with DPX (Sigma, St. Louis, MO, USA).

### Analysis of Stained Sections

The slides were examined and analyzed with a Leica microscope DMRB (Bensheim, Germany). Photographs were taken with a digital camera (Pixera Pro 600 ES; Klughammer, MarktIndersdorf, Germany) mounted on the microscope. The images were captured on a computer with Pixera Viewfinder software (Klughammer) and processed with Photoshop 7.0 (Adobe Systems, Mountain View, CA, USA). In each complete image the sharpness, contrast, and brightness were adjusted using the unsharp mask and levels adjustment tool of Photoshop until the appearance of the labeling seen through the microscope was achieved. The images were arranged and labeled with CorelDraw (version 11.0; Corel Corporation).

The dual immunofluorescence staining of selected sections was imaged with a Leica TCS SP5 laser-scanning confocal fluorescence microscope (Leica, Heidelberg, Germany). Images were taken with a 63× oil objective at a resolution of approximately 310 nm per pixel. Dual-channel imaging of Alexa 488 and Cy^3^ fluorescence was sequentially recorded at 488 nm excitation/525–550 nm emission and 564 nm excitation/555–620 nm emission. Z-series were collected at 0.31 μm optical sections taken through the section. Image stacks were processed using ImageJ (public domain, Java-based image processing program developed at the National Institutes of Health).

### Puncta Counts and Cell Perimeter Measurements

The GAD-, and GlyT2- input to ChAT-positive motoneurons of nIII and nIV was quantified by counting immunoreactive puncta along the measured length of the contour of a motoneuron with Image J as described previously (Che Ngwa et al., 2014). Only those GAD-positive/Gly-positive puncta were counted, that were in the same focal plane as the attached somata and no space was seen between them suggestive for direct synaptic inputs. The analysis of each chosen group was performed on 10 μm paraffin sections. Frozen sections from two additional cases were used as a visual control for these results. At least 22 cells in each motoneuron group were analyzed.

For the quantitative analysis of GAD inputs to MR MIF motoneurons within the C-group the respective motoneurons had been pre-labeled by a tracer injection into the MR (see “Tracer Injection Case” Section). In that case the immunocytochemical detection of the tracer was combined with immunolabeling for GAD. IR MIF motoneurons were identified on the basis of their location within the C-group. The MR MIF motoneurons lie more medially, whereas the IR-MIF motoneurons lie closer to the dorsomedial border of nIII (Tang et al., 2015).

The ratio of the number of puncta per μm of cell outline was calculated with Excel software (Microsoft 2010). The average and mean terminal density of inputs, and the standard error of the mean, were calculated for all motoneuronal subgroups, including those of the LP. Data were analyzed with the PRISM 5 software (GraphPad Prism 5, San Diego, CA, USA). Statistical analysis was performed using a one-way analysis of variance (ANOVA) followed by a Bonferroniś Multiple Comparison Test (*post hoc* test) to determine the differences between all subgroups (11 groups for the statistical analysis of GABAergic input, Figure 5; nine groups for the statistical analysis of glycinergic input Figure 8). The results were considered statistically significant at *p* < 0.05.

## Results

### Tracer Injection Case

#### Medial Rectus Muscle

Injection into the MR resulted in selective labeling of three motoneuron subgroups as described earlier and shown in Figure 1 (Büttner-Ennever et al., 2001). The A-group lies in the ventral and ventrolateral part of nIII and extends throughout the whole nIII except the most caudal part. The B-group forms a circular cell group located dorsolaterally in the caudal half of nIII. The peripheral C-group dorsomedial to the nuclear boundaries of nIII consists of MIF motoneurons and extends throughout the whole rostrocaudal length of the nIII (Figure 1).

### GABAergic Input

#### SIF Motoneurons

**Figure 2:**
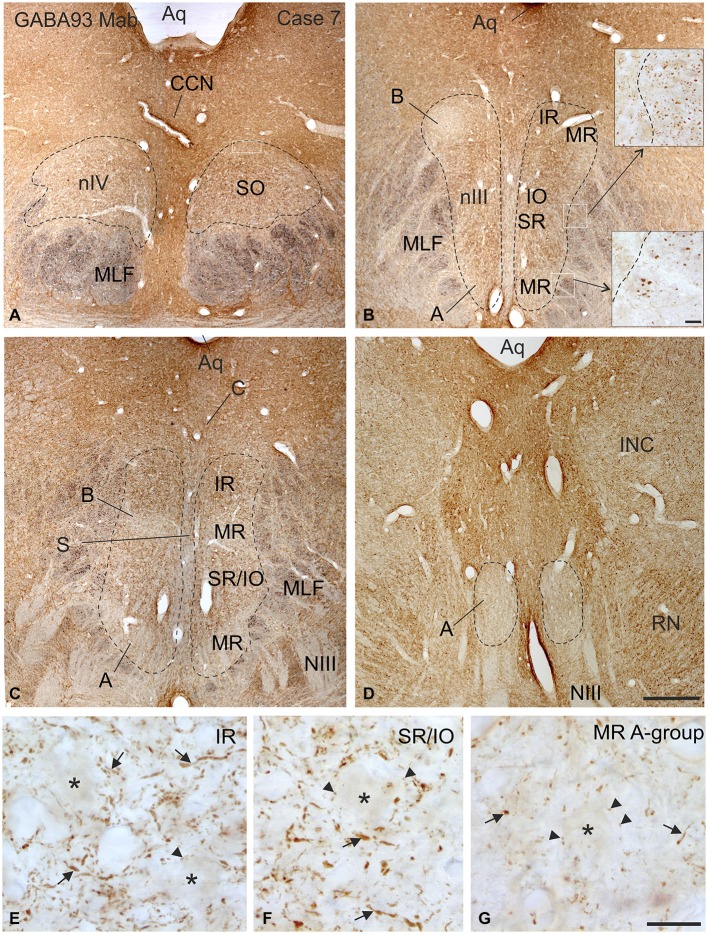
**Overview of coronal sections through the oculomotor (nIII) and trochlear nucleus (nIV) in monkey to demonstrate the immunostaining for gamma-aminobutyric acid (GABA)**. A dense labeling of GABA-positive terminals is found in nIV. **(A)** All subgroups in nIII express a similar strong GABA-immunoreactivity, except the MR A and B-group, where a weaker labeling is observed **(B–D)**. Note numerous GABAergic fibers are present in the medial longitional fascicle (MLF) next to the middle part of nIII containing motoneurons of vertically pulling eye muscles (**B**, upper inset), whereas much fewer GABA-positive fibers are found within the MLF portion adjacent to the MR A-group (**B**, lower inset). Detailed views of motoneuronal groups for vertical **(E,F)** and horizontal eye movements **(G)** reveal that most GABA immunoreactive profiles represent traversing fibers and cut axons (arrows) and only weakly stained puncta may form terminals (arrow heads) around motoneuronal somata (**E–G**, asterisks). Aq, aqueduct; CCN, central caudal nucleus, NIII, oculomotor nerve; INC, interstitial nucleus of Cajal; IO, inferior oblique muscle; RN, red nucleus; SR, superior rectus muscle; MIF, multiply innervated muscle fibers. Scale bar = 500 μm in **(D)** (applies to **A–D**); 30 μm in inset of **(B)**; scale bar = 30 μm in **(G)** (applies to **E–G**).

**Figure 3:**
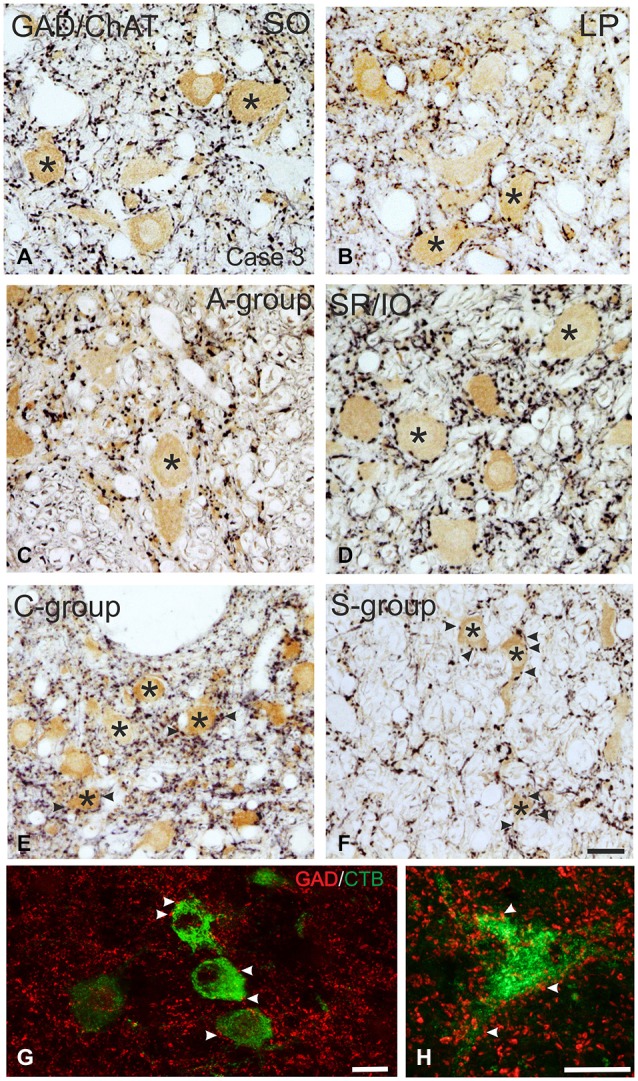
**Detailed view of coronal paraffin sections of the oculomotor (nIII) and trochlear nucleus (nIV) in the midbrain stained for glutamate decarboxylase (GAD) in black and choline acetyltransferase (ChAT) in brown (A–F)**. Numerous GAD-positive puncta outline most of the motoneuronal somata (asterisks) of the superior oblique (SO) **(A)** the levator palpebrae muscle (LP) **(B)** and the subgroup containing superior rectus (SR) and IO muscles. **(D)** In the MR subgroups fewer GAD-positive puncta are attached to the somata (**C**, asterisk), but are found in the neuropile contacting cut dendrites. A considerable number of GAD-positive puncta is found around MIF motoneurons in the C-group and S-group (**E,F**, asterisks indicate motoneurons; arrows). Detailed views of confocal images in **(G**,**H)** show tracer labeled MR MIF motoneurons (green) in the C-group that are in close association with GAD-positive (red) puncta suggestive for direct synaptic inputs (arrowheads). MIF, multiply innervated muscle fibers. Scale bar = 25 μm in **(F)** (applies to **A–F**); Scale bar = 25 μm in **(G)**; Scale bar = 25 μm in **(H)**.

**Figure 4:**
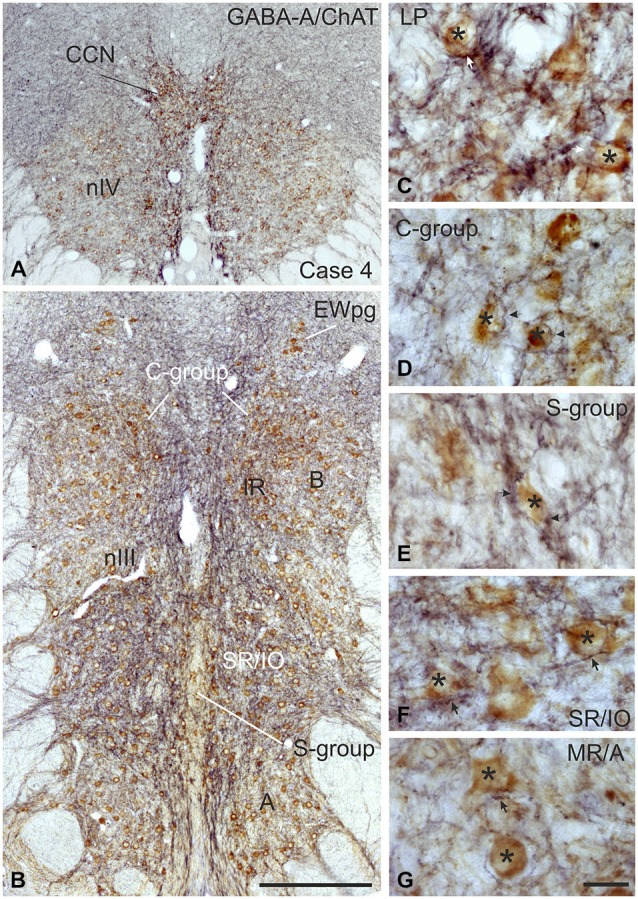
**Coronal sections of the oculomotor (nIII) and trochlear nucleus (nIV) of monkey stained for GABA-A receptor (GABA-A) in black and choline acetyltransferase (ChAT) in brown**. The overview in **(A**,**B)** shows a strong GABA-A immunoreactivity associated with the motoneurons of the levator palpebrae muscle (LP) in the CCN, with those of the superior oblique in nIV **(A)** and SR and IO motoneurons. **(B)** The detailed views demonstrate the strong GABA-A expression (arrows) around motoneurons (asterisks) of the LP and SR/IO **(C,F)** and MIF-subgroups C and S **(D,E)** compared to MR SIF motoneurons of the A-group **(G)** GABA, gamma-aminobutyric acid; MIF, multiply innervated muscle fibers; SIF, singly innervated muscle fibers. Scale bar = 400 μm in **(B)** (applies to **A,B**); 30 μm in **(G)** (applies to **C–G**).

**Figure 5:**
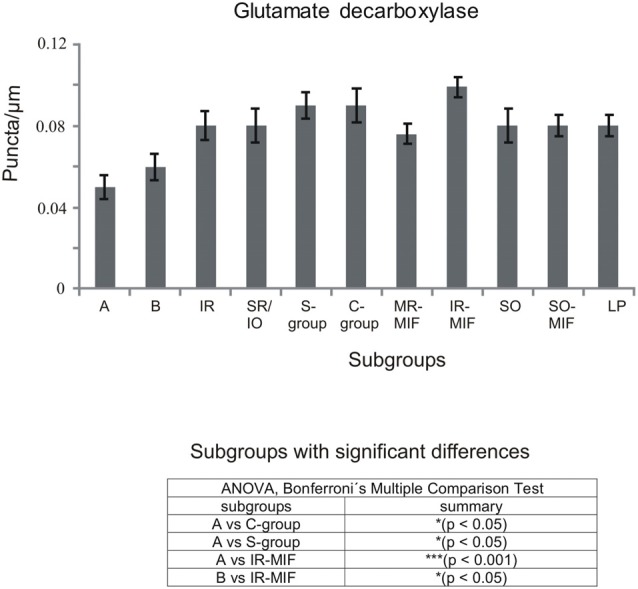
**Histogram demonstrating the quantitative analysis of glutamate decarboxylase (GAD) input to motoneurons of oculomotor (nIII) and trochlear nucleus (nIV)**. The mean terminal density of stained puncta and the standard error of the mean were calculated for all motoneural subgroups. Note a similar strong input to almost all motoneuronal subgroups in nIII and nIV. Only the motoneurons of MR (A- and B-group) receive a weaker supply by GAD-positive terminals. The table shows the results of the ANOVA and following Bonferroni ś Multiple Comparison Test.

Immunolabeling for different GABAergic markers resulted in a strong GABA- and GAD-expression within the motoneuronal subgroups of SR, IO and IR in nIII (Figures 2B,C, 3D). Similarily, the SO motoneurons in nIV and the LP motoneurons in CCN expressed strong immunoreactivity for GABA and GAD (Figures 2A, 3A,B). Visual inspection of all sections revealed a weaker GABA immunoreactivity in the MR subgroups, e.g., the ventral A-group and dorsolateral B-group, which are considered to be the SIF MR motoneurons (Figures 2B,C,D; Büttner-Ennever et al., 2001; Eberhorn et al., 2005). The weaker immunolabeling in MR subgroups was not so evident in sections immunostained for GAD (Figure 3C). The detailed views in Figure 2 demonstrate a strong GABA-expression in axons travelling within the medial longitudinal fascicle (MLF; Figures 2A–C, insets in Figure 2B) and within the motoneuronal subgroups of eye muscles mediating vertical (Figures 2E,F, arrows) and horizontal gaze (Figure 2G, arrows). The rather weak GABA-immunoreactivity in presumed nerve endings around motoneurons (Figures 2E–G, asterisks) may be one reason for the differences seen in the GAD and GABA-staining pattern (Figures 2E–G, arrowheads). Since GAD immunoreactivity was strongly expressed in nerve endings (Ottersen and Storm-Mathisen, 1984), thin paraffin sections stained for GAD were used for the quantitative analysis of GABAergic input to motoneurons (Figure 3). The counting revealed a similarly dense GAD-positive puncta supply around the somata of presumed SIF motoneurons for SR/IO, IR, SO and LP in CCN with an averaged density (AD) of 0.08 puncta/μm (Table 3; Figures 3A,B,D, 5). SIF motoneurons of MR were contacted by fewer GAD-positive puncta, with an AD of 0.05 puncta/μm for the A-group and 0.06 puncta/μm for the B-group (Table 3; Figures 3C, 5). Immunostaining for the GABA-A receptor reflected that of GAD and GABA (Figure 4) with a weaker expression within the MR subgroups (Figure 4, compare C, F to G).

**Table 3:** **Quantification of GABAergic and glycinergic input to nIV and nIII subgroups**.

GAD			Glycine		
Subgroups	Puncta/μm	SEM	Subgroups	Puncta/μm	SEM
SIF SR/IO	0.08	0.008	SIF SR/IO	0.02	0.006
SIF IR	0.08	0.007	SIF IR	0.01	0.004
SIF SO	0.08	0.008	SIF SO	0.01	0.004
LP	0.08	0.005	LP	0.15	0.015
SIF MR A	0.05	0.006	SIF MR A	0.06	0.006
SIF MR B	0.06	0.006	SIF MR B	0.07	0.006
MIF SR/IO	0.09	0.007	MIF SR/IO	0.02	0.008
MIF MR	0.07	0.005	MIF MR/IR	0.08	0.01
MIF IR	0.01	0.005			
MIF SO	0.08	0.005	MIF SO	0.02	0.01

*SEM, standard error of the mean*.

#### MIF Motoneurons

The close inspection of presumed non-twitch MIF motoneurons revealed the following picture. A high density of GAD-immunoreactive puncta and a strong immunostaining for GABA-A receptor was present in the C-group containing MR and IR MIF motoneurons (Figures 3E, 4D). This observation was clarified by the analysis of tracer-labeled MR MIF motoneurons for GAD-immunoreactivity. Numerous GAD-positive profiles were in close proximity to tracer-labeled MIF motoneurons suggesting synaptic contacts (Figures 3G,H, arrowheads). Similarily, numerous GAD-positive puncta were found around cholinergic neurons in the S-group, which represent MIF motoneurons of SR and IO (Figure 3F arrows), and in the dorsal cap of nIV containing MIF motoneurons of SO (not shown).

The quantitative analysis for the MIF motoneurons resembled the visual impression and revealed a strong supply of GAD-positive puncta for the S-group, the C-group (both 0.09 puncta/μm) and for the SO MIF motoneurons (0.08 puncta/μm; Figure 5). The analysis of the GAD input to tracer-labeled MR motoneurons in the C-group revealed an AD of 0.07 puncta/μm for the MR MIF motoneurons and 0.1 puncta/μm for IR MIF motoneurons.

To determine the differences between the different motoneuronal groups within nIII and nIV, 11 subgroups were compared to each other (see Figure 5). According to ANOVA and the subsequent Bonferroniś Multiple Comparison Test a significant difference was determined between following subgroups (Figure 5): IR MIF motoneurons received a significantly higher GAD-positive supply compared to motoneurons of the A- and B-group. Motoneurons of the C- and S-group were contacted by significantly more GAD-positive puncta compared to MR SIF motoneurons of the A-group. For more details see Figure 5.

## Immunohistochemical Localization of Glycine

### Glycine Transporter 2 (GlyT2) and Glycine-Receptor 1 (GlyR1)

#### SIF Motoneurons

**Figure 6:**
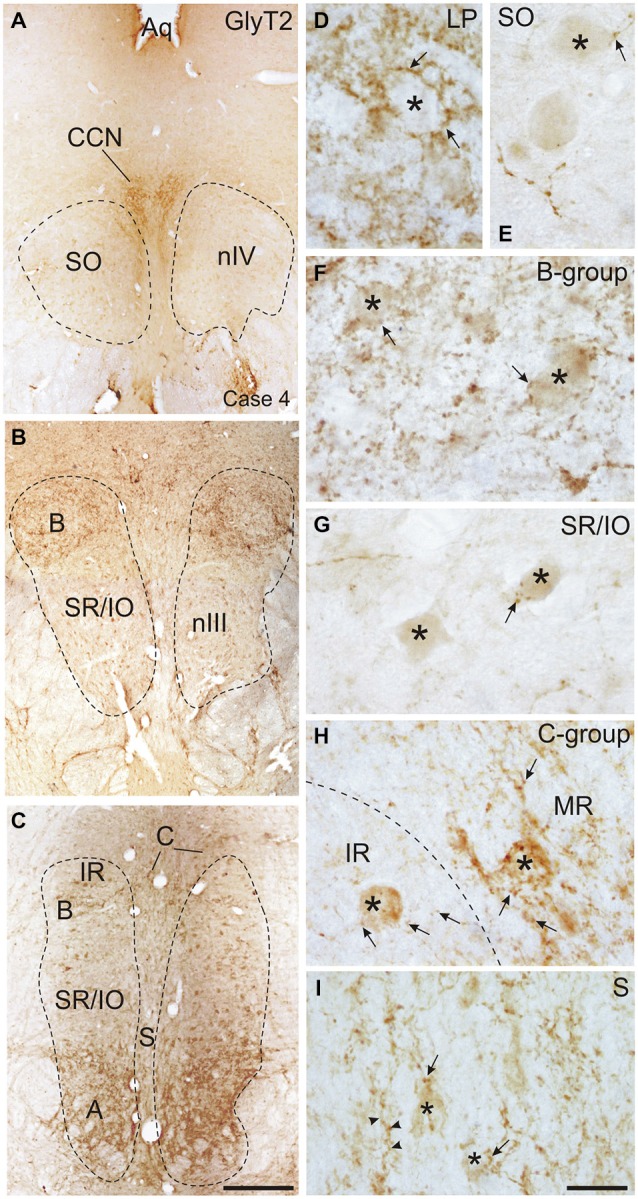
**Overviews of coronal sections through the monkey trochlear (nIV) and oculomotor nucleus (nIII) immunostained for glycine transporter 2 (GlyT2)**. A dense glycinergic input is found to levator palpebrae motoneurons (LP) in the CCN **(A,D)** and to **(A)** and **(B)** groups of the MR muscle **(B,C,F)** compared to a few GlyT2-positive fibers and puncta (arrow) scattered within nIV **(A,E**) and the superior rectus/inferior oblique (SR/IO) motoneuronal group **(B,C,G)**. **(H)** Shows a detailed view of the C-group (left side) with a MIF MR motoneuron (asterisk) covered with numerous GlyT2-positive puncta (arrows) next to a MIF inferior rectus (IR) motoneuron (asterisk) associated with only few GlyT2-positive puncta (arrows). Similarily, neurons in the S-group (asterisks) are associated with GlyT2-positive puncta and fibers (**I**, arrow). Aq, aquaeduct; IR, inferior rectus; SO, superior oblique. Scale bar = 500 μm in **(C)** (applies to **A–C**); Scale bar = 30 μm in **(I)** (applies to **D–I**).

**Figure 7:**
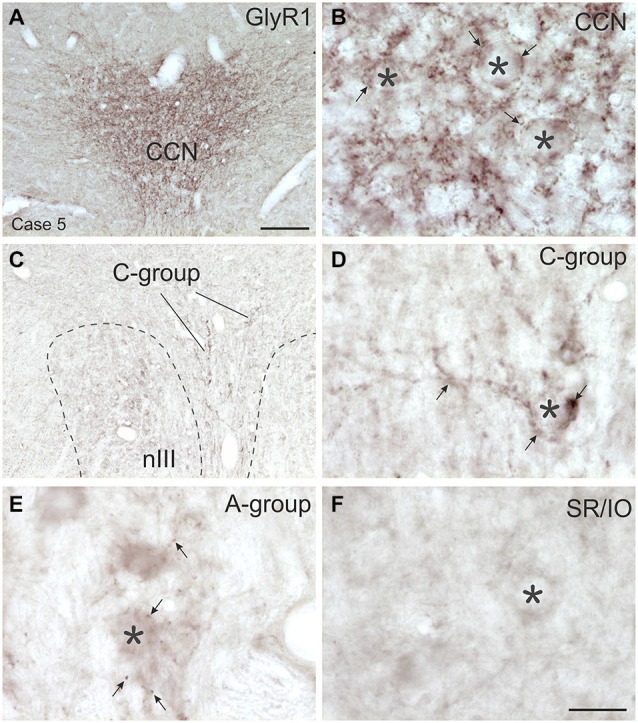
**Overviews and detailed views of immunoperoxidase labeling for glycine receptor 1 (GlyR1) in coronal sections of the oculomotor nucleus (nIII) in monkey**. The strongest GlyR1 expression is present in the CCN as puncta profiles (**A,B**, stars and arrows). Unlike the motoneurons of the superior rectus and inferior oblique subgroup (SR/IO; **F**, asterisk) the MR SIF motoneurons (**E**, asterisks) show few GlyR1-positive puncta (**E**, arrows). The MIF motoneurons in the C-group (**C**, lines; **D**, asterisk) are associated with numerous GlyR1-positive puncta (**D**, arrows). MIF, multiply innervated muscle fibers; SIF, singly innervated muscle fibers. Scale bar = 200 μm in **(A)** (applies to **A**,**C**); Scale bar = 30 μm in **(F)** (applies to **B,D–F**).

**Figure 8:**
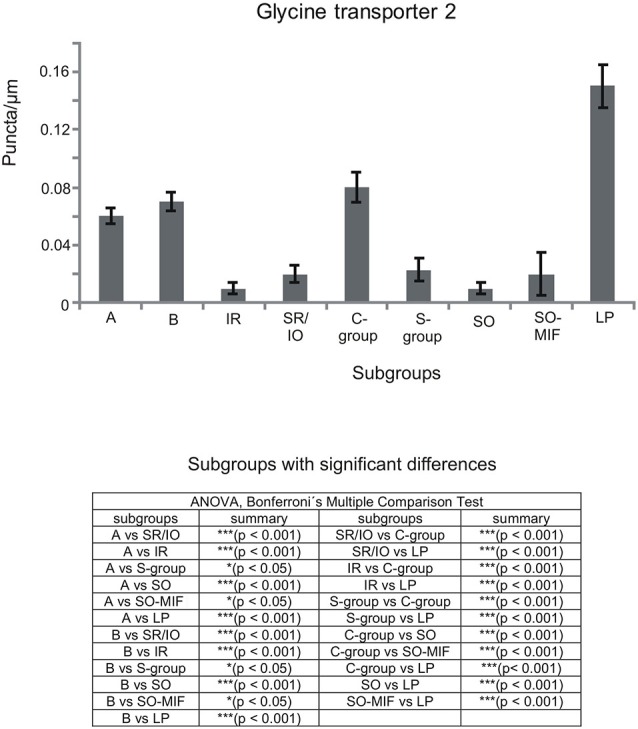
**Histogram demonstrating the quantitative analysis of the glycine transporter 2 (GlyT2) input to motoneurons in the trochlear (nIV), oculomotor (nIII) and CCN**. The glycinergic input to all motoneural groups was quantified by counting immunoreactive puncta along the measured length of the contour of a motoneuron. The mean terminal density of inputs and the standard error of the mean were calculated for all motoneural subgroups. The strongest supply by GlyT2-positive puncta is seen to levator palpebrae motoneurons (LP), and fewer puncta are found around MR motoneurons of the A-, B- and C-group. The density of GlyT2-positive puncta around motoneurons for horizontal eye movements (MR) was significant stronger compared to those for vertical eye movements. The added table shows the results of the ANOVA and following Bonferroni ś Multiple Comparison Test.

The strongest expression of glycine markers was found within the CCN. No differences in location and intensity in immunostaining were noted between GlyT2 and GlyR1 within the CCN, where the somata and proximal dendrites of LP motoneurons were completely outlined by immunoreactive puncta (Figures 6A,D, 7A,B). This was confirmed by the quantitative analysis of GlyT2 input revealing an AD of 0.15 puncta/μm (Figure 8). A strong GlyT2 expression was also found in the MR A- and B-group (Figures 6B,C,F,H). In the subgroups containing motoneurons of the vertical pulling eye muscles only few GlyT2-positive traversing fibers and GlyT2-positive puncta attached to motoneuronal somata were detected (Figures 6E,G, arrows). The GlyR1-staining pattern resembled this observation (Figures 7C,F), but revealed only a weak immunoreactivity in the MR A- and B-group as well (Figures 7C,E, arrows). The systematic quantitative analysis confirmed a dense supply of GlyT2-positive putative terminals of SIF MR motoneurons in the A-group with an AD of 0.06 puncta/μm, and 0.07 puncta/μm for the B-group (Table 3). In contrast, only a low number of GlyT2-positive puncta was found attached to IR (0.01 puncta/μm), SR/IO (0.02 puncta/μm) and SO SIF motoneurons (0.01 puncta/μm; Figures 6A,C,G, 8).

#### MIF Motoneurons

The results for the C-group MIF motoneurons were similar to those of the MR SIF motoneurons within nIII. A considerable supply of glycinergic puncta was noted to the MIF motoneurons of the MR and IR in the C-group (0.08 puncta/μm; Table 3; Figures 6C,H, arrows) also seen in immunostaining for GlyR1 (Figures 7C,D, arrows). Putative MIF motoneurons of the S-group were located between traversing GlyT2-positive fibers (Figure 6I, arrowheads), but only few puncta profiles (0.02 puncta/μm) were in contact with the cell bodies (Table 3; Figure 6I, arrows). The same observation was made for putative SO MIF motoneurons in the dorsal cap of nIV (0.02 puncta/μm; Table 3; Figure 6E, arrow). To determine the differences between the different motoneuronal groups within nIII and nIV, nine subgroups were compared to each other (see Figure 8). According to ANOVA and the subsequent Bonferroniś Multiple Comparison Test a significant difference was found between following subgroups (Figure 8): LP motoneurons in the CCN were contacted by significantly more GlyT2-positive puncta compared to MR SIF motoneurons of the A- and B-group, motoneurons of SR/IO and IR and compared to MIF motoneurons of the S-, C-group and of SO. The A- and B-group were associated with significantly more GlyT2-positive profiles compared to motoneurons of SR/IO, IR, and SO (Figures 6B,C,E,F,G). Motoneurons of the A- and B-group receive more GlyT2 inputs compared to MIF motoneurons of the S-group and of SO (Figures 6F,I). In addition, the density of the GlyT2 -positive puncta profiles to the C-group was significantly higher compared to the input to MIF motoneurons of the S-group and those of SO. The GlyT2 input to the C-group was also denser compared to that of SIF motoneurons of SR/IO, IR and SO. Thus the density of GlyT2-positive puncta around SIF motoneurons for horizontal eye movements was significantly higher compared to those for vertical eye movements. For more details see Figure 8.

### Immunohistochemical Localization of vGlut

**Figure 9:**
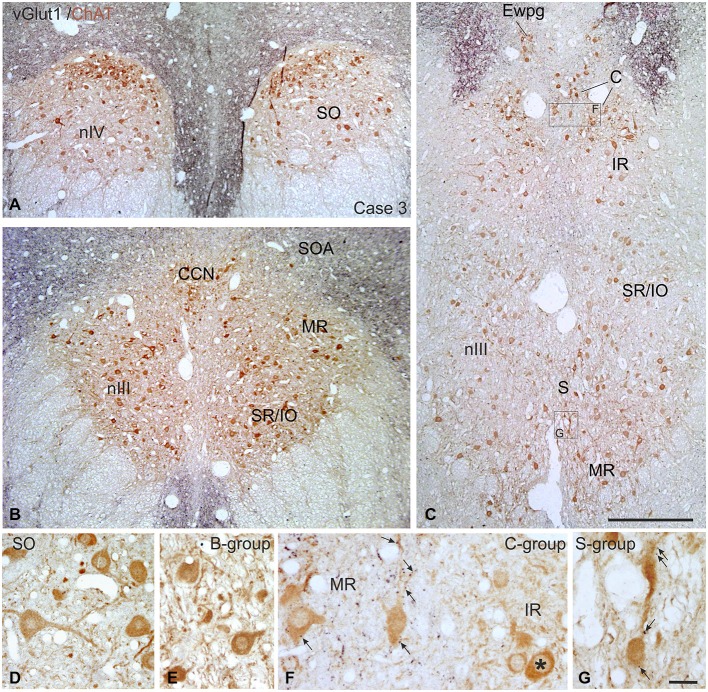
**Overviews of coronal paraffin sections through the monkey trochlear (nIV) and oculomotor nucleus (nIII) immunostained for the simultaneous detection of vesicular glutamate transporter1 (vGlut1) in black and choline acetyltransferase (ChAT) in brown (A–C)**. Note that SIF motoneuron subgroups in nIV **(A,D)** and nIII **(B,C,E)** are devoid of vGlut1-positive neuronal structures. Detailed views confirm the complete lack of vGlut1-positive puncta in nIV **(D)** and nIII with the B-group as examples **(E)**. Note that numerous vGlut1-positive puncta are attached only to MIF motoneurons in the C-group—and there confined to the MR motoneurons (**F**, arrows), but not present at IR motoneurons (**F**, asterisk). MIF motoneurons in the S-group are associated with few vGlut1-positive puncta (**G**, arrows). EWpg; preganglionic Edinger-Westphal nucleus; MIF, multiply innervated muscle fibers; SIF, singly innervated muscle fibers. Scale bar = 500 μm in **(C)** (applies to **A–C**), Scale bar = 30 μm in **(G)** (applies to **D–G**).

**Figure 10:**
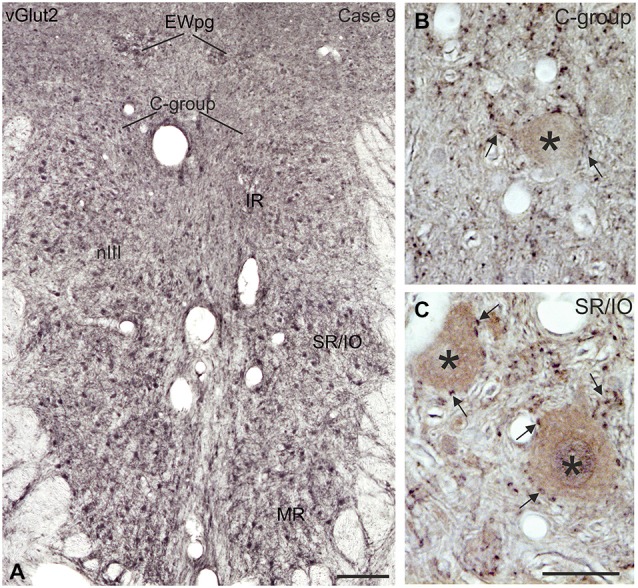
**Coronal section of the oculomotor nucleus (nIII) in monkey showing the expression of vesicular glutamate transporter 2 (vGlut2). (A)** No differences were noted in the vGlut2 puncta labeling in different motoneuronal subgroups. The detailed views in **(B**,**C)** demonstrate the similar dense input of vGlut2-positive puncta (black) around cholinergic motoneurons (brown, asterisks) in the C-group and around motoneurons of the vertically pulling superior rectus/ inferior oblique (SR/IO) muscles (**B,C**, arrows). EWpg, preganglionic Edinger-Westphal nucleus. Scale bar = 250 μm in (**A**, 30 μm in **(C)** (applies to **B,C**).

Immunolabeling for vGlut revealed that the nIII and nIV were completely devoid of vGlut1-positive terminals and neurons, except for a weak puncta labeling along the midline between both nIII (Figures 9A–C). A dense cluster of vGlut1-positive terminals was seen dorsolateral to nIII (Figure 9C). The supraoculomotor area (SOA) above nIII and the perioculomotor region around nIII contain fewer vGlut1 positive puncta (Figure 9B). At close inspection it was obvious that a considerable number of vGlut1-positive puncta was attached to putative MR MIF motoneurons in the medial C-group, but not IR MIF motoneurons (Figures 9C,F, arrows; Tang et al., 2015). Unlike for vGlut1, an even dense supply of vGlut2-positive puncta was found within nIII covering the somata of all motoneuronal subgroups (Figure 10A). Detailed views revealed a similar dense supply of vGlut2-positive puncta to MIF and SIF motoneurons as shown here for the C-group and SR/IO subgroup (Figures 10B,C).

## Discussion

The present work in part confirms previous studies on the differing inhibitory input to motoneurons subserving horizontal and vertical eye movements, respectively. It extends these findings by a quantitative analysis of the differing transmitter inputs to MIF vs. SIF motoneurons in monkey. Additionally, we showed for the first time that vGlut1-positive terminals are only associated with MIF neurons. Although direct synaptic contacts were not proven by EM studies in the present work, the close proximity between motoneurons and nerve endings suggest synaptic inputs. The results are discussed against the background of the current knowledge on premotor sources targeting MIF and/or SIF motoneurons and their transmitters.

### GABAergic Input to nIII and nIV

With the application of GABA and GAD antibodies our results confirm previous studies in monkey demonstrating that GABAergic neuronal profiles are predominantly associated with motoneurons subserving vertical eye movements (Figures 2–5; Spencer et al., 1992). Furthermore, the preferential presence of GABA-A around motoneurons of vertically pulling eye muscles is in line with the observation that postsynaptic inhibitory postsynaptic potentials (IPSPs) evoked by electrical stimulation of the labyrinth in rabbit are blocked by the GABA-A antagonist picrotoxin (Ito et al., 1970).

The strong GABAergic input to CCN may arise from the principal trigeminal nucleus, whose electrical stimulation in cat evoked IPSPs in LP motoneurons (May et al., 2012) possibly interrupting the tonic activity of LP to enable the orbicularis oculi muscle to contract during blinks (Evinger and Manning, 1993).

The reports of a GABAergic input to MR motoneurons are most controversial for different species, but may depend on differences in the methods and applied antibodies. In accordance with the present results a moderate supply of GABAergic terminals was noted in the MR subdivisions in monkey and cat using immunohistochemistry in frozen sections (Spencer et al., 1989; Spencer and Baker, 1992). However, studies applying postembedding GABA staining in semithin sections did not detect a significant difference of GABAergic input to tracer-labeled MR motoneurons compared to the other subdivisions in nIII, in cat and rabbit (de la Cruz et al., 1992; Wentzel et al., 1996), similar to the quantification of GAD-positive inputs in thin paraffin sections in the present study. In human, the number of GAD-positive profiles within the putative MR subgroups even exceeds that of motoneuron groups involved in vertical gaze. This may indicate an evolvement of inputs related to vergence, which is particularly prominent in human (see below; Che Ngwa et al., 2014). The finding of a considerable GABAergic input to the C- and S-groups confirms previous observations in monkey (Ying et al., 2008).

#### Glycinergic Input to nIII

The considerable supply of GlyT2-positive nerve endings to MR subdivisions A and B resembled the labeling pattern of glycine-positive afferents in nIII of previous reports in monkey (Figures 6–8; Spencer and Baker, 1992; Poyatos et al., 1997). The similar distribution pattern of GlyT2-positive nerve endings in human nIII served there to identify the homolog MR subgroups (Che Ngwa et al., 2014). In cat, glycinergic terminals were found in all motoneuron subgroups except the MR subdivisions (Spencer and Baker, 1992). In rabbit, a glycinergic input was noted to all subdivisions in the nIII including the MR region (Wentzel et al., 1996), but may colocalize with GABA (Wentzel et al., 1993). Based on current knowledge about MIF motoneuron organization the glycinergic terminals around the midline are now considered to target the IO and SR MIF motoneurons within the S-group (Büttner-Ennever et al., 2001; Wasicky et al., 2004), rather than the SR/IO SIF motoneurons (Spencer and Baker, 1992; Spencer et al., 1992).The previously described association of GlyT2 with LP motoneurons in the CCN was confirmed and is in line with a strong expression of glycine receptor 1 seen here. The saccadic omnipause neurons were shown as one possible glycinergic source to CCN (Horn and Büttner-Ennever, 2008).

Functionally, glycine is similar to GABA as it increases chloride conductance and evokes, therefore, IPSPs. Consequently, the likelihood that the postsynaptic cell reaches the threshold for firing an action potential reduces. The colocalization of glycine and GABA in afferent inputs to MR motoneurons may indicate a co-release of both transmitters (Wentzel et al., 1993). As shown for abducens motoneurons it is possible that GABA-A and glycine receptors are distributed differently at somatal or dendritic membranes (Lorenzo et al., 2007), which may play a role in tuning the IPSPs (Russier et al., 2002).

Glycine can also serve as co-agonist with glutamate at postsynaptic N-methyl-D-aspartate (NMDA) receptors (Johnson and Ascher, 1987). It is an open question as to whether the glycinergic input to the MR subdivisions in nIII, which showed a weak GlyR expression, serve as an inhibitory transmitter, or as a co-agonist of excitatory glutamatergic afferents, e.g., from internuclear neurons (INT) in nVI or ventral lateral vestibular nucleus (LVN; see below; Nguyen and Spencer, 1999).

#### Glutamatergic Input to nIII and nIV

This is the first description of the expression pattern of vGlut associated with eye muscle motoneurons. VGluts selectively package glutamate into synaptic vesicles and mediate glutamate transport and therefore are used as markers for glutamatergic neuronal profiles (Takamori et al., 2000; Fremeau et al., 2001, 2004; Zhou et al., 2007; for review: El Mestikawy et al., 2011). Whereas SIF and MIF motoneurons in nIII and nIV receive a dense vGlut2-positive supply, a specific vGlut1-positive input was found only to MIF motoneurons, mainly those of MR (Figure 9F).

Transient responses of glutamate transmission are mediated through ionotropic NMDA and non-NMDA (AMPA and kainate) receptors, whereas more persistent responses are mediated by metabotropic G-protein coupled receptors (Dingledine et al., 1999). Thereby AMPA receptors convey the fast component of postsynaptic responses, whereas NMDA receptors mediate long lasting slower postsynaptic responses. Both, NMDA-receptors and AMPA receptors (GluR4 subunit), are only expressed in SIF, but not in MIF motoneurons in monkey (Ying et al., 2008). This may indicate that SIF motoneurons participate in the fast and slow components of the postsynaptic response to glutamate. This is in line with *in vitro* studies on rat oculomotor neurons showing that smaller motoneurons with low-recruitment threshold currents have higher input resistances and exhibit tonic firing—as assumed for MIF motoneurons and whose firing pattern remains essentially unmodified by glutamate application (Torres-Torrelo et al., 2012). The phasic-tonic firing of larger motoneurons—such as SIF motoneurons—with lower input resistances and with high recruitment threshold currents, is strengthened by glutamate and could provide strong muscle contractions for (saccadic) eye movements (Torres-Torrelo et al., 2012).

#### Premotor Sources and their Association with Transmitters

##### Secondary vestibulo-ocular neurons (only SIF)

A well-established input to nIII and nIV arises from the vestibular nuclei subserving the vertical angular vestibulo-ocular reflex (VOR; for review: Büttner-Ennever and Gerrits, 2004; Straka and Dieringer, 2004; Highstein and Holstein, 2006; Goldberg et al., 2012). Primary afferents from the anterior and posterior canals activate secondary vestibular neurons in the magnocellular parts of the medial vestibular nucleus (MVNm) and superior vestibular nucleus (SVNm), which in turn send contralateral excitatory and ipsilateral inhibitory projections to the respective motoneurons of agonists and antagonists in nIII and nIV (Graf and Ezure, 1986; Iwamoto et al., 1990; Graf et al., 1997; Goldberg et al., 2012).

Extracellular tracer injections into SVNm or MVNm and single cell reconstructions of identified up and down position-vestibular-pause neurons indicated that secondary vestibular neurons target only SIF motoneurons, but not MIF motoneurons in the S- or C-group (McCrea et al., 1987; Wasicky et al., 2004). This is in line with the lack of transneuronally labeled secondary vestibular neurons in SVNm or MVNm after the injection of rabies virus into the myotendinous junction of eye muscles, from where only MIF (not SIF) motoneurons were retrogradely filled (Ugolini et al., 2006).

##### Glutamate and aspartate

Glutamate and/or aspartate are widely accepted as the major excitatory neurotransmitter of the secondary vestibular neurons (Demêmes and Raymond, 1982; for review: McElligott and Spencer, 2000) and may reflect at least one portion of the vGlut2-positive input to nIII and nIV. This is in line with the presence of numerous neurons expressing vGlut2 mRNA, but only few expressing weak vGlut1 mRNA signals in rat vestibular nuclei (Hisano et al., 2002; Zhang et al., 2011). The excitatory glutamatergic second-order vestibular inputs onto abducens neurons act through AMPA receptors (Straka and Dieringer, 1993). This pattern may apply to SIF motoneurons in nIII and nIV, as indicated by their high expression of AMPA receptors (GluR1, 2, 3 and 4) in human and monkey (Williams et al., 1996; Ying et al., 2008). Both, glutamate and NMDA, produce a depolarization of NMDA receptors primarily located at dendrites, but are not associated with the excitatory second-order vestibular input to oculomotor motoneurons (Durand and Gueritaud, 1990).

Another source of glutamatergic input to nIII is conveyed by the ascending tract of Deiters (ATD; Nguyen and Spencer, 1999; Büttner-Ennever and Gerrits, 2004; Holstein, 2012). It originates from secondary vestibular neurons in the ventral MVN and the ventral LVN that receive excitatory inputs from the ipsilateral labyrinth, and target MR A and B subgroups in the ipsilateral nIII (not C-group; McCrea et al., 1987). The ATD carries head velocity signals, which are modulated by utricular inputs and the viewing distance of visual targets to generate disconjugate vergence eye movements (Reisine et al., 1981; Chen-Huang and McCrea, 1998; Angelaki, 2004). Postembedding immunostaining indicated that ATD afferents may use glutamate as a transmitter, whereas the additional aspartate-labeling was attributed to the metabolic pool (Nguyen and Spencer, 1999). This glutamate/aspartate projection targets primarily somata and proximal dendrites of MR motoneurons via synapses with asymmetric densities and spheroidal vesicles, the classical features of excitatory synapses (Nguyen and Spencer, 1999). These projections may well be included in vGlut2-positive afferents of the present study.

##### GABA

Electrophysiological and pharmacological studies have identified GABA as a major inhibitory transmitter of vertical secondary vestibulo-ocular neurons in different species (for review: McElligott and Spencer, 2000; Straka and Dieringer, 2004; Sekirnjak and du Lac, 2006). These inhibitory projections arise in the SVN and MVN, and target the ipsilateral SO and IR, or SR and IO motoneurons (Holstein, 2012), predominantly at their somata and proximal dendrites (Spencer and Baker, 1992; Wentzel et al., 1995). A fraction of the GABAergic fibers in the MLF seen in the present study may represent the inhibitory connections of the vertical VOR (see also Spencer et al., 1989; for review: Goldberg et al., 2012).

##### Non-secondary vestibulo-ocular connections (SIF and MIF)

Additional projections to nIII and nIV arise from non-secondary vestibular neurons, which are not directly activated by primary afferents from the semicircular canals (Goldberg et al., 2012). They include the dorsal y-group, which receives disynaptic inputs from vertical canal afferents (Blazquez et al., 2000), and projects to SIF *and* MIF motoneurons of SR and IO in the contralateral nIII and to IR and SO motoneurons on the ipsilateral side (Carpenter and Cowie, 1985; Wasicky et al., 2004). Electrical stimulation studies suggest that the y-group may be part of cerebellar pathways for vertical smooth-pursuit eye movements (Chubb and Fuchs, 1982). At least a subpopulation of non-secondary vestibular neurons from the parvocellular MVN (MVNp) and the dorsal y-group, which target only motoneurons of upward moving eye muscles, contains calretinin (CR; Ahlfeld et al., 2011; Zeeh et al., 2013). These CR terminals were found to be excitatory (Zeeh et al., 2013) and may contribute to the vGlut-positive input to SIF and MIF neurons seen here (Figures 9, 10).

##### Abducens internuclear neurons (only SIF)

Another excitatory input to MR neurons arises from abducens INT, which carry a head-velocity and head position signal (burst, and burst-tonic) providing the neuroanatomical basis for conjugate horizontal eye movements (for review: Highstein and Holstein, 2006). In cat the tracer-labeled synaptic endings of abducens INTs within the contralateral MR subgroup were shown to express immunoreactivity for glutamate and aspartate (Nguyen and Spencer, 1999). The differing spatial location of glutamatergic afferents from INTs and the ATD indicates that the more proximal location of ATD synaptic input onto MR neurons may reduce the threshold for activation by the more distally located glutamatergic input from INTs during conjugate horizontal eye movements (Delgado-Garcia et al., 1986; Nguyen and Spencer, 1999). This may be used to reduce the synaptic delay from INT input to MR to ensure conjugacy of horizontal eye movements.

Since tracer injections into nVI result in afferent labeling of all MR subgroups including the MIF motoneurons in the C-group (Wasicky et al., 2004), a strong glutaminergic input via this pathway must be anticipated (Nguyen and Spencer, 1999). The more distal INT terminals on MR motoneurons are thought to act through NMDA and non-NMDA (AMPA) receptors at the same postsynaptic site (Brodin and Shupliakov, 1994). This arrangement is consistent with the known somato-dendritic distribution of NMDA and non-NMDA receptors on both, second-order vestibular (Cochran et al., 1987) and other extraocular motoneurons (Durand et al., 1987; Durand and Gueritaud, 1990; Durand, 1991; Straka and Dieringer, 1993).

##### RIMLF and INC (SIF, SIF and MIF)

Another monosynaptic input to SIF motoneurons of vertically pulling eye muscles originates from burst neurons in the rostral interstitial nucleus of the medial longitudinal fasciculus (RIMLF) and the interstitial nucleus of Cajal (INC) encoding vertical and torsional saccades (Moschovakis et al., 1991a,b; Horn and Büttner-Ennever, 1998; Kokkoroyannis et al., 1996; for review: Horn, 2006). Tracer-labeled afferents from RIMLF to nIII express glutamate and aspartate (Spencer and Wang, 1996). Both amino acids act on NMDA receptors, and in addition, glutamate acts on non-NMDA receptors and may mediate different components of the postsynaptic response, and could thereby contribute to vGlut2 inputs.

GABAergic premotor neurons in the dorsomedial part of the RIMLF in cat (Spencer and Wang, 1996) and in INC in monkey (Horn et al., 2003) may monosynaptically inhibit the motoneurons of anatogonistic eye muscles during up or downward saccades as shown by intracellular recording studies in cat (Sugiuchi et al., 2013). Since lesions of INC result in vertical gaze-holding deficits with a head-tilt (Büttner et al., 2002) the INC is considered to function as velocity-to-position integrator of vertical eye movements (for review: Fukushima and Kaneko, 1995). This function may be provided by premotor burst-tonic and tonic neurons that receive a burst signal from RIMLF and project monosynaptically to motoneurons of vertical pulling eye muscles to transmit eye position signals (Dalezios et al., 1998; Horn and Büttner-Ennever, 1998; Sugiuchi et al., 2013). It is reasonable to assume that these premotor fibers target SIF *and* MIF motoneurons, as indicated from tract-tracing after small biocytin injections into INC in monkey (Kokkoroyannis et al., 1996). The differing projections from RIMLF and INC to SIF and MIF motoneurons conform to the concept that SIF motoneurons are driven only from burst neurons in RIMLF and INC to generate the eye movement, whereas the burst-tonic and tonic input from INC targets also MIF motoneurons for gaze holding. Taken together it can be reasoned that premotor excitatory burst and burst-tonic neurons in RIMLF and INC provide a glutamatergic input to the motoneurons of vertical eye movers, and thereby may form a portion of the vGlut2-positive input to nIII and nIV.

##### Prepositus nucleus

A strong projection from the prepositus nucleus (PPH) to the ipsilateral MR subgroup has been demonstrated (Baker et al., 1977; McCrea and Baker, 1985; Belknap and McCrea, 1988; for review: McCrea and Horn, 2006). Correlation of the neural activity of antidromically activated PPH neurons, with the resultant ipsilateral eye movements and contralateral head movements, suggest an inhibitory action of this projection (Delgado-García et al., 1989), although excitatory projections to nIV may also be present (Baker et al., 1977). Since no GABAergic projection from PPH to nIII has been found in monkey (Carpenter et al., 1992), the inhibition may be transmitted via glycine, as is the case for the abducens nucleus (Spencer et al., 1989). Whether the GlyT2-positive input to MR motoneurons in this study may represent inhibitory projections from the PPH remains to be studied (Figures 6–8).

##### Inputs to only MIF motoneurons: Premotor sources and association with transmitters

The most selective transmitter-related input was found from vGlut1-positive afferents to MR MIF motoneurons including their dendrites, which reach up into the supraoculomotor area (SOA) approaching the preganglionic neurons in the preganglionic Edinger-Westphal nucleus (EWpg) controlling pupillary constriction and lens accommodation for the near response (Tang et al., 2015; for review: McDougal and Gamlin, 2015). Thereby the SOA is a well-suited target for premotor inputs controlling the near response as suggested by the abundance of synaptic contacts at the distal dendrites of MR MIF motoneurons compared to only few synapses targeting their somata and proximal dendrites (Erichsen et al., 2014). One source may arise from “near response neurons” in the SOA that increase their activity during convergence and can be antidromically activated from MR subgroups (Judge and Cumming, 1986; Mays et al., 1986; Zhang et al., 1991, 1992).

In monkey, a selective premotor input only to MIF motoneurons was first described from the pretectum (Gamlin and Clarke, 1995; Büttner-Ennever et al., 1996; Wasicky et al., 2004). This includes the nucleus of the optic tract, which projects specifically to MIF motoneurons of nIII and nIV, and the olivary pretectal nucleus, which targets primarily pupil-related preganglionic neurons in the rostral EWpg via excitatory synapses (Gamlin and Clarke, 1995; Büttner-Ennever et al., 1996; Wasicky et al., 2004; Sun and May, 2014a, b). Another possible source is the central mesencephalic reticular formation (CMRF), which is associated with horizontal and vertical conjugate eye movements (Waitzman et al., 1996; Wang et al., 2013). Recent tracer studies in monkey demonstrated a strong projection from premotor neurons in the CMRF to the SOA including the C-group and the EWpg (Bohlen et al., 2015). This projection is bilateral and, if excitatory, may participate in the control of vergence and the near triad (Bohlen et al., 2015). Whether glutamatergic neurons in the SOA, in the pretectal nuclei or the CMRF give rise to the selective vGlut1 input to the somata or dendrites of MIF motoneurons, remains to be studied (Fujiyama et al., 2003).

## Conclusion

In conclusion the exclusive vGlut1 input to MIF motoneurons and the higher density of GABA/glycinergic inputs to MR MIF motoneurons in the C-group compared to SIF motoneurons within nIII confirm the concept that SIF and MIF motoneurons receive different inputs from premotor areas involved in different functions: SIF motoneurons in generating eye movements, MIF motoneurons in gaze holding including vergence in the near response (Wasicky et al., 2004; Büttner-Ennever, 2006; Ugolini et al., 2006). MIF neuron groups were shown to contain also the cell bodies of palisade endings inserting at the myotendinous junction of extraocular muscles (Lienbacher et al., 2011; Zimmermann et al., 2011). But up to date it is not clear, whether they form a separate population of presumed sensory neurons or are part of motoneurons giving rise to the multiple innervation and palisade endings at non-twitch muscle fibers (Lienbacher and Horn, 2012).

Although all SIF motoneurons are involved in similar tasks exhibiting similar firing behavior during eye movments, the functional significance of differences in the chemical properties of premotor inputs—as seen for the specific expression of calretinin in excitatory premotor pathways for upgaze—is unclear (Zeeh et al., 2013). Transmitter inputs may not only convey a specific postsynaptic reponse, but may modulate the excitability of the motoneurons, for example by opening chloride channels conveyed by GABA and glycine (Lorenzo et al., 2007). Recent *in vivo* studies in rat demonstrated that the firing properties of motoneurons in nIII (tonic and phasic discharge) as function of recruitment threshold current and cell size can be modified by glutamatergic input (Torres-Torrelo et al., 2012). Based on their findings from *in vitro* studies of rat nIII motoneurons superfused with GABA, the authors propose that motoneuron firing rates are essentially driven by transient neurotransmission of different transmitters. Thereby this transient mechanism could act as a modulation system refining the output of the motoneurons (Torres-Torrelo et al., 2014).

## Conflict of Interest Statement

The authors declare that the research was conducted in the absence of any commercial or financial relationships that could be construed as a potential conflict of interest.

## Abbreviations

AD: averaged density
AMPA receptors: α-amino-3-hydroxy-5-methyl-4-isoxazolepropionic acid receptor
ATD: ascending tract of Deiters
CCN: central caudal nucleus
ChAT: choline acetyltransferase
CMRF: central mesencephalic reticular formation
CR: calretinin
CTB: Cholera toxin subunit B
EWpg: preganglionic Edinger-Westphal nucleus
GABA: gamma-aminobutyric acid
GABA-A: GABA-A receptor
GAD: glutamate decarboxylase
GlyR: glycine receptor
GlyT: glycine transporter
INC: interstitial nucleus of Cajal
INT: internuclear neurons
IO: inferior oblique muscle
IPSP: inhibitory postsynaptic potential
IR: inferior rectus muscle
LP: levator palpebrae muscle
LR: lateral rectus muscle
LVN: lateral vestibular nucleus
MIF: multiply-innervated muscles fibers
MLF: medial longitudinal fasciculus
MR: medial rectus muscle
MVN: medial vestibular nucleus
MVNm: MVN magnocellular part
MVNp: MVN parvocellular part
nIII: oculomotor nucleus
nIV: trochlear nucleus
NMDA: N-methyl-D-aspartate
nVI: abducens nucleus
PPH: prepositus nucleus
RIMLF: rostral interstitial nucleus of the medial longitudinal fasciculus
SIF: singly-innervated muscles fibers
SO: superior oblique muscle
SOA: supraoculomotor area
SR: superior rectus muscle
SVN: superior vestibular nucleus
SVNm: SVN magnocellular part
TBS: Tris buffered saline
vGlut: vesicular glutamate transporter
VOR: vestibulo-ocular reflex
WGA-HRP: wheat germ agglutinin conjugated to horseradish peroxidase.
